# Amplification of 9p24.1 in diffuse large B-cell lymphoma identifies a unique subset of cases that resemble primary mediastinal large B-cell lymphoma

**DOI:** 10.1038/s41408-019-0233-5

**Published:** 2019-08-30

**Authors:** Yucai Wang, Kerstin Wenzl, Michelle K. Manske, Yan W. Asmann, Vivekananda Sarangi, Patricia T. Greipp, Jordan E. Krull, Keenan Hartert, Rong He, Andrew L. Feldman, Matthew J. Maurer, Susan L. Slager, Grzegorz S. Nowakowski, Thomas M. Habermann, Thomas E. Witzig, Brian K. Link, Stephen M. Ansell, James R. Cerhan, Anne J. Novak

**Affiliations:** 1 Division of Hematology, Mayo Clinic, Rochester, MN USA; 2 Department of Health Sciences Research, Mayo Clinic, Jacksonville, FL USA; 3 Department of Health Sciences Research, Mayo Clinic, Rochester, MN USA; 4 Division of Laboratory Genetics and Genomics, Mayo Clinic, Rochester, MN USA; 5 Division of Hematopathology, Mayo Clinic, Rochester, MN USA; 6 Division of Hematology, Oncology, and Bone & Marrow Transplantation, University of Iowa, Iowa City, IA USA

**Keywords:** B-cell lymphoma, Cancer genetics

## Abstract

Copy number alterations (CNAs) of 9p24.1 occur frequently in Hodgkin lymphoma, primary mediastinal large B-cell lymphoma (PMBCL), primary central nervous system lymphoma, and primary testicular lymphoma, resulting in overexpression of PD-L1 and sensitivity to PD-1 blockade-based immunotherapy. While 9p24.1 CNA was also reported in diffuse large B-cell lymphoma (DLBCL), little is known about its molecular or clinical significance. In this study, we analyzed the prevalence of 9p24.1 CNA in newly diagnosed DLBCL and examined its association with PD-L1, PD-L2, and JAK2 expression, clinical characteristics, and outcome. We found that 10% of DLBCL cases had CNA of 9p24.1, with 6.5% gains, and 3.5% amplifications. Only the cases with a 9p24.1 amplification had high levels of PD-L1, PD-L2, and JAK2 expression. Gains or amplifications of 9p24.1 were associated with a younger age and the ABC/non-GCB subtype. Compared with DLBCL cases without 9p24.1 CNA, the cases with a 9p24.1 amplification had a trend of better event-free survival. Furthermore, the amplification cases had a gene expression and mutation profile similar to those of PMBCL. Our data suggest that amplification of 9p24.1 identifies a unique subset of DLBCL with clinical and molecular features resembling PMBCL that may be amenable to PD-1 blockade-based immunotherapy.

## Introduction

Diffuse large B-cell lymphoma (DLBCL) is the most common type of non-Hodgkin lymphoma (NHL). Immunochemotherapy with R-CHOP is an efficacious treatment, and has remained the standard of care for two decades^1–5^. However, ~40% of patients are refractory to or relapse after R-CHOP. Although some patients can be salvaged with second-line chemotherapy, the majority will eventually succumb to the disease^6^. Gain of insight on the mechanisms that drive DLBCL pathogenesis and impact response to therapy is key to improving the outcome of patients with this disease. Molecular characterization of DLBCL provides critical biologic information, and based on distinct gene expression profiles, DLBCL can be divided into the germinal center B-cell-like (GCB) and the activated B-cell-like (ABC) subtypes^7,8^. The ABC subtype is associated with more aggressive disease and worse clinical outcome^9^. Genetic characterization of DLBCL also has important clinical implications. A subset of DLBCL cases have translocations of *MYC*, *BCL-2*, and/or *BLC-6*, and these genetic alterations dictate significant aggressiveness and dismal clinical outcome^10,11^. More recently, comprehensive genomic studies have further defined unique genetic subtypes of DLBCL, each associated with distinct pathogenesis mechanisms and clinical outcomes^12–16^.

Copy number alterations (CNA) of 9p24.1, including chromosomal amplification, gain, polysomy, or translocation, are one of the hallmarks of classical Hodgkin lymphoma (cHL)^17,18^, and also frequently occur in extranodal large B-cell lymphomas, such as primary mediastinal large B-cell lymphoma (PMBCL)^17,19–22^, primary central nervous system lymphoma (PCNSL)^22^, and primary testicular lymphoma (PTL)^22^. These genomic alterations can lead to increased expression of key genes in the region, including *PDCD1LG2/PD-L2*, *CD274/PD-L1*, and *JAK2*^17,18^. Programmed death-ligand 1 (PD-L1) and PD-L2 signal through the programmed cell death protein 1 (PD-1) receptor on T cells and serve as an immune checkpoint to negatively regulate T-cell-mediated immunity^23^. A variety of malignancies take advantage of this signaling axis to evade immune surveillance by upregulating expression of PD-L1 through diverse mechanisms^23,24^. Immune checkpoint inhibitors targeting PD-1 such as nivolumab or pembrolizumab have proven to be efficacious in treating relapsed and/or refractory cHL^25–28^, and have also been shown to be effective in PMBCL, PCNSL, and PTL^29,30^.

CNA of 9p24.1 have also been detected in DLBCL, albeit at lower frequencies^17,19,22,31^. Green et al. first reported 9p24.1 amplification in 4 of 18 DLBCL cell lines^17^, and additional studies have since reported 9p24.1 amplification in up to 6% of DLBCL patients^19,20,22,31,32^. In addition, copy number gain and translocations have been reported in up to 16 and 4% of DLBCL patients, respectively^19,31,32^. To date, little is known about the impact of 9p24.1 CNA on the clinical outcome of DLBCL patients. In this study, we analyzed the prevalence of 9p24.1 CNA in untreated newly diagnosed DLBCL and examined its association with PD-L1, PD-L2 and JAK2 expression, clinical characteristics, and outcome. Supporting published data, our study finds that copy number gains in 9p24.1 occur in 10% of DLBCL cases. Our data also show that not all copy number gains are biologically comparable; only those cases with a 9p24.1 amplification have high levels of PD-L1, PD-L2, and JAK2 expression. Furthermore, we find that the 9p24.1 amplification cases have clinical and molecular features that resemble PMBCL and represent a unique subset of DLBCL that might be amenable to immune checkpoint blockade therapy.

## Materials and methods

### Study population

This study was approved by the Mayo Clinic Institutional Review Board. A total of 199 patients with newly diagnosed DLBCL from May 2002 to September 2012 were included in this study; cases with an initial diagnosis of PMBCL were excluded. All patients were treated with standard immunochemotherapy and followed prospectively through the Molecular Epidemiology Resource (MER) of the University of Iowa/Mayo Clinic Lymphoma Specialized Program of Research Excellence (SPORE). Full details of this prospective cohort study of lymphoma outcomes have been previously published^33^. All patients provided written consent forms at enrollment into MER for their clinical samples to be analyzed. Patients were followed every 6 months for the first 3 years, and annually thereafter. Disease progression, relapse, unplanned re-treatment after initial immunochemotherapy and death from any cause were verified through medical record review. Cell of origin (COO) on available cases was determined by gene expression profiling (GEP, *n* = 33)^8^, Lymph2Cx assay (NanoString, *n* = 97)^34^, or the Hans algorithm (*n* = 49)^35^. Baseline clinical characteristics of the 199 DLBCL patients in this study are shown in Supplementary Table 1.

### Copy number analysis

In total, 57 of the cases were analyzed for CNAs using raw whole-exome sequencing (WES) data files^36^. The remaining cases (*n* = 142) were processed at the Mayo Clinic Cytogenetics Lab using the molecular inversion probe OncoScan™ FFPE Assay Kit (Affymetrix, Santa Clara, CA, USA). For the latter samples, DNA was extracted from formalin-fixed, paraffin-embedded (FFPE) DLBCL tumors using the QIAamp DNA FFPE Tissue Kit (Qiagen GmbH, Hilden, Germany) in the Mayo Biospecimens Accessioning and Processing Core. Prior to isolation, tumor blocks were reviewed by a Mayo Clinic hematopathologist, tumor areas were circled, and four 1 mm cores were used for DNA isolation. The minimum tumor purity for study was 30%. DNA quantity was measured using the Qubit fluorometer (Thermo Scientific, Waltham, MA, USA) instrument. Raw WES bam files and OncoScan OSCHP files were analyzed using Nexus Copy Number 9.0 software (Biodiscovery, El Segundo, USA). The data interpretation and copy number calling were done using the human reference genome GRCh37/hg19. Files were analyzed using the Nexus FASST2-Segmentation algorithm, which is based on a Hidden Markov Model approach for calling genetic event. Nexus standard configuration for gain calling thresholds were used. For WES files, thresholds were set at a log_2_ratio above 0.18 for gain and 0.6 for amplification. Thresholds for OncoScan files were set at log_2_ratio above 0.1 for gain and 0.7 for amplification. All files have been reviewed for correct diploid calling according to the log_2_ratio and B-Allele frequency of each sample, and have been adjusted if calling was not correct. Copy number events which were smaller than 100 kb were removed from the analysis. For Oncoscan files, gains that include a minimum of 50 probes and losses that include a minimum of 25 probes were called. Furthermore, to remove false positive alterations, we filtered against parameters provided by Nexus that include 89852 normal structural variation listed in the Database of Genomic Variants (DGV)^37^ so that those calls, which have the same size and region listed as normal CNA, are automatically removed.

### Fluorescence in situ hybridization (FISH)

Tissue microarray (TMA) slides containing patient DLBCL samples were used for the FISH studies. Home-brew bacterial artificial chromosome clones RP11-980L14, RP11-927H16, and CTD-2506A8 covering the *JAK2* locus (locations shown in Supplementary Fig. 1) labeled with SpectrumOrange dUTP (Abbott Molecular, Chicago, IL, USA) and chromosome 9 centromere labeled with SpectrumGreen dUTP (Abbott Molecular, Chicago, IL, USA) were used as target and control probes, respectively. The probe set was applied to individual TMA slides, hybridized, and washed according to standard FISH protocol. Fifty nuclei were analyzed for each sample. Normal indicates two target signals and two control signals, gain was defined as a target:control probe ratio of >1 but ≤2 and an average target probe signal < 6, and amplification was defined as a target:control probe ratio >2 or a target probe signal ≥6. Polysomy was defined as a target:control probe ratio of 1 and an average target probe signal >2 but <6.

### RNAseq

RNA-Seq was performed at the Broad Institute, and data were available on 31 cases used in this study. The data were processed and analyzed using the Mayo Clinic RNA sequencing in house analysis pipeline MAPRSeq (v2.1.1)^38^. Quality control was performed by using RSeQC^39^. Briefly, 50-bp paired-end reads were aligned to human reference genome 37 by using Tophat (v2.1.0), and the counts per gene and per exon were summarized using HTSeq^40^ using ENSEMBL gene v78. The log_2_ transformed reads per million per kb (RPKM) were generated for gene differential expression analyses.

### Immunohistochemistry (IHC) staining

IHC staining was performed on TMA slides using a PD-L1 antibody (rabbit clone SP263, Ventana Medical Systems, Tucson, AZ, USA) and a PAX5 antibody (clone DAK-Pax5, Dako, Santa Clara, CA, USA) to identify B cells. Staining was performed on the Ventana Benchmark XT platform with on-line deparaffinization, HIER (Heat Induced Epitope Retrieval) with Ventana CC1 buffer for 64 min, and primary antibody incubation at 37 °C for 16 minutes. Antigen-antibody reactions were visualized using the Ventana OptiView DAB IHC Detection Kit. The percentage of PD-L1-positive neoplastic large B cells and the intensity of staining (1–weak staining, 2–moderate staining, 3–strong staining) were quantified by an experienced hematopathologist (R.H.). The H-score was calculated by multiplying the pertange of PD-L1-positive lymphoma cells by the intensity of staining.

### Gene expression profiling

Gene expression profiling (GEP) of DLBCL tumors using Affymetrix HG U133 plus 2.0 arrays was performed at the Broad Institute (GSE98588), and data were available on 38 cases used in the study. The array raw intensity data were pre-processed using Robust Multiarray Averaging (RMA) method for background correction, quantile normalization, and median polish probe set expression value summary^41^. The log_2_ transformed and RMA processed expression values were used in the analyses.

### Statistical analysis

Comparison of quantitative data between groups was done by Student’s *t* test or one-way ANOVA test (assuming normal distribution). Association of 9p24.1 CNA with clinical and pathological factors was analyzed by Chi-square test. Event-free survival (EFS) was defined as time from diagnosis to progression or relapse, unplanned re-treatment after initial immunochemotherapy, or death from any cause. EFS between groups were compared using the Kaplan–Meier method and the log-rank test. Statistical analysis was done in SPSS (V22, IBM, Armonk, NY, USA). All reported *P-*values were two-sided, and a *P-*value < 0.05 was considered statistically significant.

## Results

### Frequency of 9p24.1 CNA in DLBCL

**Fig. 1:**
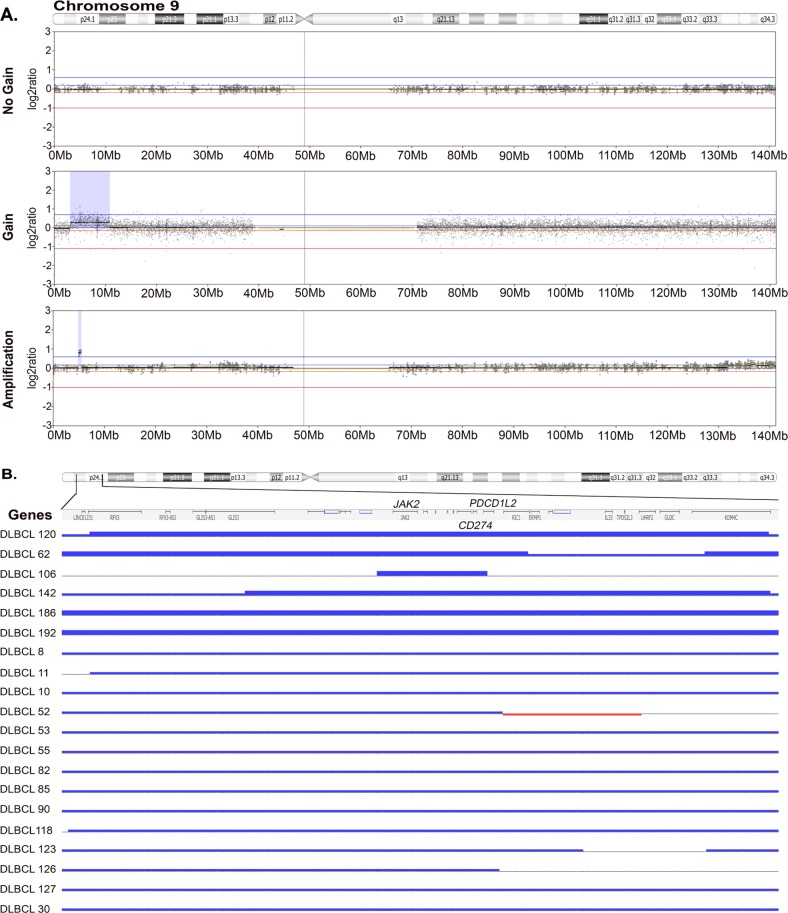
9p24.1 copy number gain and amplification in DLBCL. **a** Representative log_2_ratio plots of chromosome 9 showing no gain (*n* = 179), gain (*n* = 14), and amplification (*n* = 6) of 9p24.1. Highlighted blue region indicates gain or amplifation region. **b** Detailed view of 9p24.1 in the 20 cases with a gain or amplification. Thick (*n* = 6) and thin (*n* = 14) blue lines indicate amplification and gain, respectively

Genome-wide chromosomal copy number analysis of human cancers is technically challenging due to several inherent variables including sample type, tissue preservation method, and quality of DNA. In the clinical setting, isolation of DNA for genomic studies often requires use of FFPE samples. The OncoScan^®^ FFPE Assay Kit (Affymetrix) was the first microarray designed specifically for use with degraded DNA, which is often found in FFPE tissue. The Oncoscan array utilizes SNP probes to provide copy number as well as allele frequency information. To gain insight on DLBCL-specific CNAs, we used OncoScan to characterize the copy number landscape of DLBCL tumors (*N* = 142, dbGAP submission in progress). An additional cohort of tumors was analyzed using WES data (*n* = 57)^36^. The data files from WES and OncoScan were analyzed using the Nexus Copy Number software program. The copy number landscape of DLBCL analyzed by WES or OncoScan is shown in Supplementary Fig. 2A. Nineteen cases had data available by both WES and OncoScan and gene level CNA calls were highly concorrdant (Supplementary Fig. 2B), suggesting that both methods yield comparable data. As an additional validation step, we compared our CNA data, including 31 high frequency gains and losses, with publically available data on DLBCL CNA^16^. Again, the concordance in calling CNAs was significant (*P* < 0.003; Supplementary Fig. 2C). Using the combined data set of 199 tumors, we determined the frequency of 9p24.1 CNA in DLBCL. Only cases who had a *PDCD1LG2/PD-L2*, *CD274/PD-L1*, and *JAK2* gain were considered. Overall, the analysis identified three subgroups of CNA, cases that had no gain of 9p24.1 (*n* = 179), cases that had a gain (*n* = 14), and cases that had an amplification (*n* = 6). Representative examples of each is shown in Fig. 1a. A detailed view of the 9p24.1 chromosomal region and a summary of CNA in the 20 cases with a gain or amplification are shown in Fig. 1b. All 20 patients had a gain at the *CD274/PD-L1* and *JAK2* loci; 19 had a gain at the *PDCD1LG2/PD-L2* loci, and 1 had partial gain that included exon 1 and exon 2. A summary of the 20 cases with 9p24.1 CNA and the analysis performed on each case is shown in Supplementary Table 2.

Overall, these studies suggest that OncoScan is a highly sensitive method for detection of DLBCL CNA in clinical samples and that 10% of DLBCL have a copy number gain or amplification at 9p24.1.

### Confirmation of 9p24.1 CNA in DLBCL by FISH

**Fig. 2:**
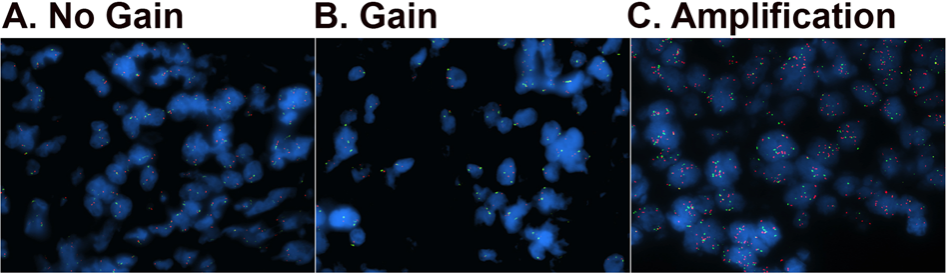
Validation of 9p24.1 copy number gain and ampilfication by FISH. Tissue microarray (TMA) slides containing patient DLBCL samples were used for the FISH studies. Fifty nuclei were analyzed for each sample. Normal indicates two target signals and two control signals, gain was defined as a target:control probe ratio of 1–2 and an average target:control probe signal < 6, and amplification was defined as a target:control probe ratio > 2 or a target probe signal ≥ 6. Representative images of no gain (**a**), gain (**b**), and amplification (**c**) of 9p24.1 are shown

The identification of two subgroups of CNA patterns by WES/OncoScan was intriguing, and we next wanted to validate these findings using a separate approach. Therefore, we performed FISH analysis to confirm our results (Fig. 2). Of the 20 gain or amplification cases, 9 had tissue available for FISH analysis. Eleven cases that had no gain reported by Nexus were also included in the study. As shown in Supplementary Table 2, of the four amplified cases, FISH confirmed an amplification in all four (*JAK2* signals > CEN9 signals with a ratio >2 and an average of ≥ 6 *JAK2* signals), confirming our prior results. For the five gain cases, FISH identified two cases with a gain (*JAK2* signals > CEN9 signals with a ratio >1 but <2 and an average of < 6 *JAK2* signals), two cases were polysomy (*JAK2* signals = CEN9 signals and an average *JAK2* signal between 2 and 6), and one case was called an amplification. Visual review of the FISH data on this specific case suggested that only a subset of cells had an amplification which was not detected by OncoScan. In addition, we noted that this case, as well as all of the other amplification cases, had a focal gain at 9p24.1 (<1.5 × 10^7^ base pairs, Supplementary Fig. 3). Therefore, for the remaining analysis, this case was considered an amplification. None of the no gain cases were called a gain or an amplification by FISH. Together these studies suggest that copy number and FISH analysis can identify unique subsets of 9p24.1 CNA, gain (*n* = 13, 6.5%), and amplification (*n* = 7, 3.5%), in DLBCL.

### Expression of PD-L1, PD-L2, and JAK2

**Fig. 3:**
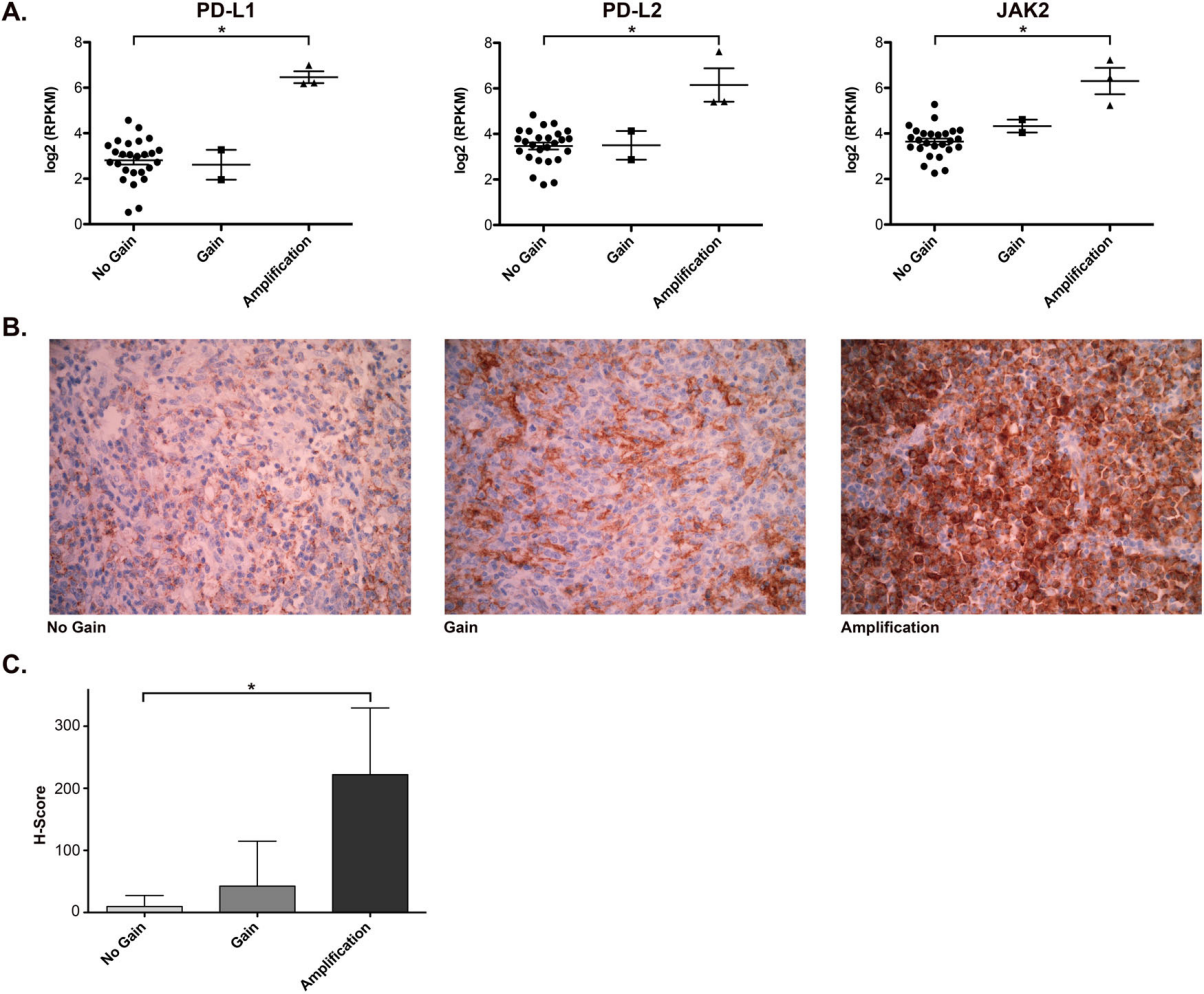
Expression of PD-L1, PD-L2 and JAK2. **a** Quntification of mRNA expression of PD-L1, PD-L2, and JAK2 determined by RNAseq in DLBCL with no gain (*n* = 26), gain (*n* = 2), or amplification (*n* = 3) of 9p24.1. Error bars represent standard deviation. **b** Representative images of DLBCL tissue stained with anti-PD-L1 in 9p24.1 no gain, gain, and amplification cases. **c** The H-score of PD-L1 staining in lymphoma cells in DLBCL with no gain (*n* = 10), gain (*n* = 4), or amplification (*n* = 5) of 9p24.1. Error bars represent standard error of the mean

Selective amplification of the 9p24.1 region is associated with increased expression of the *CD274/PD-L1*, *PDCD1LG2/PD-L2*, and *JAK2* genes at both the transcript and protein levels^17^. To determine whether 9p24.1 CNA in DLBCL is associated with overexpression of these genes, we examined expression of PD-L1, PD-L2, and JAK2 in DLBCL patients with or without 9p24.1 CNA. Using available RNASeq data on 31 of the cases used for CNA analysis (3 amplifications, 2 gains, and 26 no gains), we examined the expression of *CD274/PD-L1*, *PDCD1LG2/PD-L2*, and *JAK2*. As shown in Fig. 3a, patients with an amplification of 9p24.1 had significantly higher mRNA levels of PD-L1 (log_2_RPKM, mean ± SD 6.46 ± 0.45 vs 2.81 ± 0.94, *P* < 0.001), PD-L2 (log_2_RPKM, mean ± SD 6.15 ± 1.27 vs 3.47 ± 0.77, *P* < 0.001), and JAK2 (log_2_RPKM, mean ± SD 6.30 ± 1.00 vs 3.64 ± 0.68, *P* < 0.001) compared with those with no gain of 9p24.1. The two patients with a 9p24.1 gain had similar PD-L1, PD-L2, and JAK2 mRNA levels compared with those with no gain, although the patient number was small. These results suggest that only amplification of 9p24.1 leads to increased expression of the key genes contained in this region, including *CD274/PD-L1*, *PDCD1LG2/PD-L2*, and *JAK2*.

We next measured expression of PD-L1 by immunohistochemistry in 19 of the cases used for CNA analysis (Fig. 3b). The samples were stained for PAX5 and PD-L1, and the DLBCL cells were identified by their morphology and PAX5 staining. The percentage of PD-L1-positive lymphoma cells and the intensity of PD-L1 staining were quantified and summarized graphically in Fig. 3c. Cases with no gain of 9p24.1 (*n* = 10) had little to no staining of PD-L1; in contrast, the majority of the amplification cases (*n* = 5) had strong staining of PD-L1 compared with the no gain controls (mean 74% vs 4% positive, *P* < 0.001; mean H-score 222 vs 10, *P* < 0.001). The 9p24.1 gain cases (*n* = 4) had variable staining of PD-L1 that was not significantly increased over the controls (mean 15% vs 4% positive, *P* = 0.188; mean H-score 43 vs 10, *P* = 0.183). Together these data suggest that high expression of PD-L1 correlates with chromosomal amplification of 9p24.1 and that chromosomal gains in this region are less likely to result in significantly increased PD-L1 mRNA and protein expression.

### Correlation of 9p24.1 CNA with clinical variables

To determine whether 9p24.1 CNA correlates with clinical features in DLBCL, we compared clinical characteristics of DLBCL patients with or without a 9p24.1 gain or amplification (as determine by both OncoScan and FISH). The average age of patients with 9p24.1 gain (57.2 ± 13.3) or amplification (51.4 ± 20.2) was younger than those with no gain of 9p24.1 (63.8 ± 11.8; *P* = 0.008, one-way ANOVA). As shown in Table 1, more patients with 9p24.1 gain or amplification trended towards being 60 or younger (*P* = 0.062). Sex, ECOG performance status, LDH level, number of extranodal sites, Ann Arbor stage, and the International Prognostic Index (IPI) score did not differ among the three groups. Compared with no gain of 9p24.1, cases with a 9p24.1 gain or amplification were about twice as likely to be the activated B-cell-like (ABC)/non-germinal center B-cell-like (non-GCB) subtype (*P* = 0.019).

**Table 1:** Clinical characteristics of DLBCL patients by 9p24.1 copy number status

	9p24.1 No gain	%	9p24.1 Gain	%	9p24.1 Amplification	%	Chi-square *P-*value
Age							0.062
≤60	62	34.6	7	53.8	5	71.4	
>60	117	65.4	6	46.2	2	28.6	
Sex							0.176
Male	110	61.5	9	69.2	2	28.6	
Female	69	38.5	4	30.8	5	71.4	
ECOG PS							0.443
<2	158	88.8	10	76.9	6	85.7	
≥2	20	11.2	3	23.1	1	14.3	
Missing	1						
LDH							0.260
Normal	81	50.3	2	22.2	3	50.0	
Elevated	80	49.7	7	77.8	3	50.0	
Missing	18		4		1		
Extranodal sites							0.77
≤1	145	81.0	11	84.6	5	71.4	
>1	34	19.0	2	15.4	2	28.6	
Ann Arbor stage							0.386
I–II	72	40.2	5	38.5	1	14.3	
III–IV	107	59.8	8	61.5	6	85.7	
IPI score							0.920
0–1	57	34.3	3	27.3	3	42.9	
2	51	30.7	3	27.3	1	14.3	
3	43	25.9	3	27.3	2	28.6	
4–5	15	9.0	2	18.2	1	14.3	
Missing	13		2				
Cell of origin							0.019
GCB	99	66.9	3	33.3	2	28.6	
ABC/non-GCB	49	33.1	6	66.7	5	71.4	
Unclassifiable	13		2				
Missing	18		2				

### Association of 9p24.1 CNA with clinical outcome

**Fig. 4:**
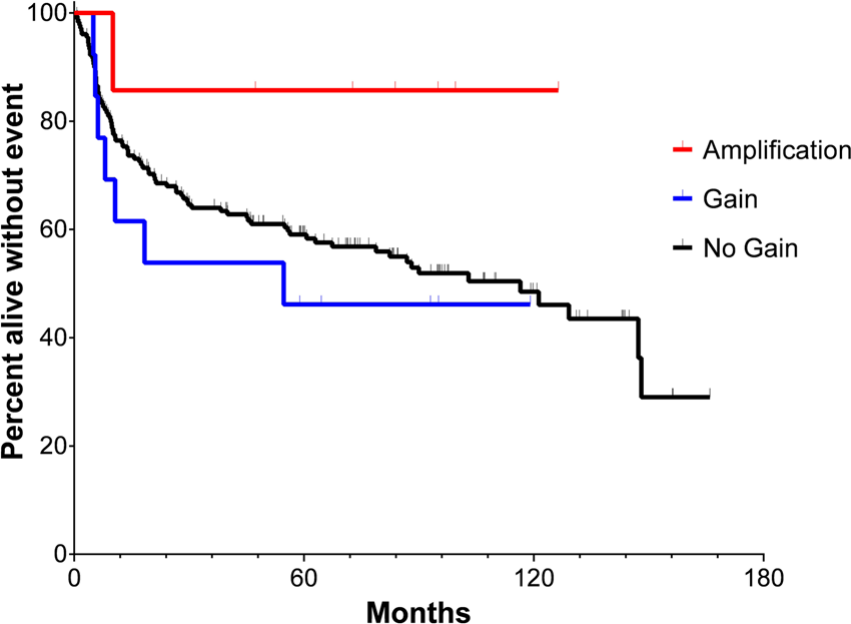
EFS of DLBCL patients with or without 9p24.1 CNA. Kaplan–Meier curves of EFS of patients with amplification (*n* = 7), gain (*n* = 13), and no gain (*n* = 179) of 9p24.1 are shown. Log-rank test was used for comparison of EFS between groups (amplification vs no gain, median EFS not reached vs 116.5 months, *P* = 0.138; gain vs no gain, median EFS 54.7 vs 116.5 months, *P* = 0.513)

In newly diagnosed cHL, 9p24.1 amplification was associated with worse progression-free survival (PFS) following treatment with the Stanford V chemotherapy regimen plus modified involved field radiation (IFRT)^18^. Therefore, we examined whether 9p24.1 CNA affected clinical outcome of newly diagnosed DLBCL following standard immunochemotherapy (such as the R-CHOP regimen) (Fig. 4). The median follow-up for the entire cohort was 95.0 months. There were a total of 94 events and 73 deaths. Patients with 9p24.1 amplification had a trend of better EFS compared with those with no gain of 9p24.1 (median EFS not reached vs 116.5 months, 2-year EFS rate 85.7% vs 68.5%, 5-year EFS rate 85.7% vs 59.1%, *P* = 0.138). In contrast, patients with 9p24.1 gain had numerically worse EFS compared with those with no gain of 9p24.1 (median EFS 54.7 vs 116.5 months, 2-year EFS rate 53.8% vs 68.5%, 5-year EFS rate 46.2% vs 59.1%, *P* = 0.513), although the difference was not statistically significant. These results suggest that 9p24.1 amplification and gain may have distinct impacts on the clinical outcome of patients with newly diagnosed DLBCL.

### Molecular characteristics of DLBCL with a 9p24.1 amplification

**Fig. 5:**
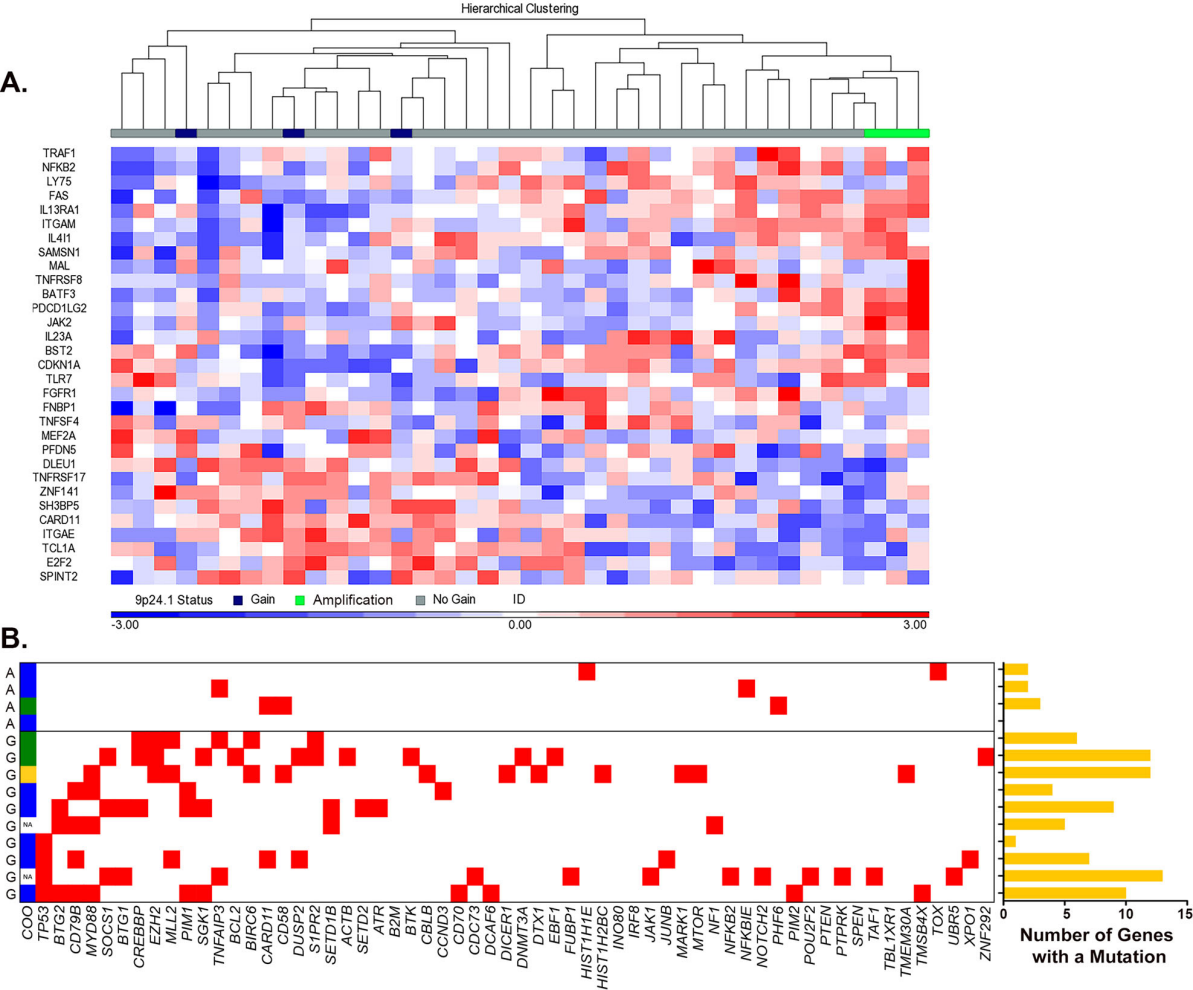
Molecular characteristics of DLBCL cases with 9p24.1 amplification. **a** Hierarchical clustering of gene expression data from DLBCL cases with no gain (*n* = 32), gain (*n* = 3), or amplification (*n* = 3) of 9p24.1 using a PMBCL GEP signature. **b** Somatic gene mutation profiles of reported DLBCL driver genes in cases with gain (G, *n* = 10) or amplification (A, *n* = 4) of 9p24.1. A red box indicates a mutation in the gene listed. COO calls were reported as ABC/non-GCB (blue), GCB (green), and unspecified (yellow). The right panel graph shows the number of reported DLBCL driver genes that have a mutation in each sample

Analysis of the clinical data on the 9p24.1 amplification cases identified a notable pattern: they were young, predominantly female (71.4%), and 86% patients were event-free 24 months after frontline standard immunochemotherapy. These clinical features resemble those of PMBCL, which also tends to occur in younger females and respond well to frontline immunochemotherapy. In addition, up to 75% of PMBCL cases are reported to have 9p24.1 amplification^17,19,20,22^. Clinical review of the seven amplification cases did not reveal a diagnosis of PMBCL. Although 5/7 had mediastinal lymph node involvement, none had a predominant mediastinal mass on PET/CT. Biopsy sites on the seven cases were variable across samples and included lymph node (*n* = 3), spleen (*n* = 2), lung (*n* = 1), and hip bone and soft tissue (*n* = 1). A recent study by Yuan et al.^42^ described the pathologic and clinical characteristics of DLBCLs that have PMBCL gene expression profiles^43^ and their findings suggest that PMBCL can present at nonmediastinal sites without mediastinal involvement. Based on these findings, we analyzed GEP data from 38 cases from our cohort and looked for expression of the PMBCL gene signature^43^. As shown in Fig. 5a, the 9p24.1 amplified cases (*n* = 3) clustered together and had a GEP consistent with the PMBCL GEP signature reported in the literature^43^. In contrast, the 9p24.1 gain cases (*n* = 3) were intermixed with the no gain cases (*n* = 32), none of which had the PMBCL GEP signature.

PMBCL also differs from ABC and GCB subtypes of DLBCL in somatic mutation profiles. ABC DLBCLs frequently have mutations in *MYD88*, *CD79B*, and *CARD11*, while GCB DLBCLs often have mutations in *CREBBP*, *EZH2*, *TNFRSF14*, and *BCL2*^13,44^. On the other hand, genes frequently mutated in PMBCL include *STAT6*, *TNFAIP3*, *SOCS1*, *CD58*, and *CIITA*^44^. To determine whether the 9p24.1 amplification cases had PMBCL-like mutation signature, we analyzed WES data from 9p24.1 CNA cases (*n* = 14; 10 with gain and 4 with amplification). Among the 150 DLBCL driver genes reported in the literature^14^, we detected mutations in 60 genes (Fig. 5b). Overall, the mutation rate was lower (*P* = 0.01) in the four amplification cases compared with the gain cases (shown graphically in Fig. 5b). Among the ten gain cases, all had at least one mutation in the DLBCL driver genes *TP53*, *CD79B, MYD88, CREBBP*, or *EZH2*. In contrast, none of the amplification cases had a mutation in these genes. Two amplification cases had a mutation of *TNFAIP3* and *CD58*, respectively, both of which occur frequently in PMBCL. Collectively, these data suggest that the DLBCL cases with amplification of 9p24.1 had molecular and genetic features similar to PMBCL.

## Discussion

Copy number alterations of 9p24.1 are commonly found in cHL and also occur in PMBCL, PCNSL, and PTL. The resulting increased expression of PD-L1 and PD-L2 make these lymphomas susceptible to immune checkpoint inhibitors targeting the PD-L1/PD-1 pathway. In this study, we used a novel OncoScan platform to characterize the DLBCL CNA landscape and identified cases with a 9p24.1 alteration at a frequency of 10%. However, only those cases with a 9p24.1 amplification have high levels of PD-L1, PD-L2, and JAK2 expression, consistent with a prior study^19^. Our study also suggests that 9p24.1 amplification cases have clinical and molecular features that resemble PMBCL and identifies a unique subset of DLBCL that might be amenable to checkpoint blockade therapy.

The 9p24.1 CNA frequency of 10% detected by WES and OncoScan in our study is within the range of reported frequencies (0 to 19%) in prior studies. The differences across studies are likely due to different sample sizes, detection techniques, and reporting criteria. Shi et al. used a PCR-based Taqman copy number assay to detect copy gain (>2.2) of *PD-L2*, and found 9 of 12 PMBCL but none of the 9 DLBCL patients had *PD-L2* gain^20^. Twa et al. used a FISH assay to analyze genomic alterations involving the *PD-L1/PD-L2* locus and the frequency of gain (3–4 signals in >20% nuclei) and amplification (≥5 signals in >20% nuclei) were 14 and 3%, respectively, in 134 DBLCL patients^19^. Georgiou et al. also used a FISH assay and detected 12% gain (3–4 copies/cell) and 3% amplification (≥5 copies/cell) of the *PD-L1/PD-L2* locus in 190 DLBCL samples^31^. Ansell et al. analyzed 9p24.1 in a fraction of patients enrolled in the CheckMate 139 study (nivolumab for relapsed/refractory DLBCL), and reported 3% amplification (target:control signal ratio ≥ 3) and 16% of gain (target:control signal ratio > 1 but <3)^32^. Chapuy et al. conducted a high-density single-nucleotide polymorphism (HD-SNP) array to define genetic alternations, and reported 9p24.1 gain in 11 (6%) of 180 DLBCL patients^22^. In our study, we also performed FISH in a subset of patients and verified the copy number status detected by WES and OncoScan. The 9p24.1 amplification cases all showed amplification by FISH, and the frequency of amplification (3.5%) detected in our study is consistent with the frequencies of amplification (3%) in two prior studies using FISH^19,22^. In a more recent study by Chapuy et al.^16^, 9p24.1 amplifications were identified in 5% of DLBCL and gains 11%, supporting our findings and further suggesting that OncoScan is a sensitive method for delineation of 9p24.1 alterations. While our study is one of the largest to date, 199 cases remain a relatively small sample size. In particular, only 7 amplification and 13 gain cases were eventually available for further molecular characterization, and future validation of our results will be important.

In our cohort, the cases with 9p24.1 gain were young and predominantly the ABC/non-GCB subtype. The mechanism of association with a younger age is unclear, but it is possible that 9p24.1 gain is an early event in DLBCL. The association of 9p24.1 gain with the ABC/non-GCB subtype was consistent with a previous report^31^, as well as a few other studies that reported an association of PD-L1 expression with the non-GCB subtype in DLBCL^45–47^. The 9p24.1 gain cases had slightly higher expression of PD-L1 by IHC compared with no gain cases (15% vs 4%), and trended toward a worse EFS following frontline immunochemotherapy. In a previous Japanese study, 34 (12.5%) of 273 DLBCL patients were PD-L1^+^ (≥ 30% cells positive for PD-L1 and PAX5 by IHC), which were associated with the non-GCB subtype and had worse overall survival (OS) compared with PD-L1^−^ patients^47^. In another recent study, Cheng et al. analyzed data from Gene Expression Omnibus (GEO) and also reported that higher PD-L1 expression (*n* = 104) was associated with a shorter OS in a cohort of 414 patients^48^. The association with the ABC/non-GCB subtype could be one of the explanations of the worse outcome in DLBCL with 9p24.1 gain. Compared with the GCB subtype, the ABC/non-GCB subtype of DLBCL is more aggressive and has an inferior outcome following frontline therapy. In addition, the activation of the PD-L1/PD-1 pathway in the gain cases may also contribute to worse clinical outcome by suppression of immune surveillance.

The patients with an amplification of 9p24.1 in our cohort are of particular interest. They were young, predominantly female, and were nearly all event-free after standard immunochemotherapy after a median follow-up close to 8 years. They had a PMBCL-like GEP signature, and carried somatic mutations that were seen frequently in PMBCL (*TNFAIP3* and *CD58*) instead of those often seen in ABC or GCB DLBCL (*TP53*, *CD79B, MYD88, CREBBP*, and *EZH2*). These clinical and molecular features suggest that the DLBCL cases with 9p24.1 amplification resemble PMBCL. Previous studies did not differentiate DLBCL cases with a lower or higher level of copy number gain, e.g., gain vs amplification. In this setting, our novel findings have important clinical implications. While a higher expression of PD-L1 was reported to be associated with adverse clinical outcome, similar to our gain cases, those cases with a particularly strong PD-L1 expression by RNASeq or IHC driven by a 9p24.1 amplification may be genetically and biologically unique. Further molecular characterization could be considered in these cases, especially in young female patients, since they may have molecular features that resemble PMLBCL and have more favorable clinical outcome with frontline therapy.

Detection of 9p24.1 gains or amplifications in DLBCL patients has important clinical implications. Patients with relapsed or refractory (R/R) DLBCL may benefit from immunotherapy targeting PD-1/PD-L1 if they harbor 9p24.1 gains or amplifications. Although objective response was seen in 4 of 11 R/R DLBCL patients in a phase Ib study of nivolumab^49^, the objective response rate (ORR) was lower (3% in ASCT-ineligible and 10% in those who failed ASCT) in a larger phase 2 study with nivolumab in R/R DLBCL (CheckMate 139)^32^. The benefit of PD-1/PD-L1 inhibitors is likely going to be limited in unselected R/R DLBCL patients. It is possible that patients with 9p24.1 gains or amplifications would respond much better to PD-1/PD-L1 blockade therapy than those without, and it would be interesting to study the efficacy of PD-1/PD-L1 blockade in this specific population. It will also be important to determine the clinical significances of 9p24.1 amplification vs gain. Our data, combined with studies from PMBCL and cHL, suggest a potential benefit for cases that have an amplification. Second, addition of a PD-1 or PD-L1 inhibitor to standard immunochemotherapy can potentially improve the outcome of ABC DLBCL. Note that 18% (11/60) of ABC/non-GCB DLBCL patients had 9p24.1 gain or amplification in our study. The presence of 9p24.1 combined with high PD-L1 expression in ABC is a plausible mechanism to escape T-cell mediated anti-lymphoma activity. Immunomodulation with lenalidomide is a promising strategy to improve the outcome of ABC DLBCL^50^. Combining a PD-1 or PD-L1 antibody with standard immunochemotherapy for frontline treatment of ABC DLBCL may lead to improved outcome. Phase 2 clinical trials combining pembrolizumab (NCT02541565) or durvalumab (NCT03003520) with chemotherapy in untreated DLBCL are currently ongoing. It would be valuable to perform biomarker analyses in these studies to see whether 9p24.1 CNA is predictive for benefit of addition of these immune checkpoint inhibitors.

## Supplementary information

Supplementary Material
