# Supplementary material for: Amplification of 9p24.1 in diffuse large B-cell lymphoma identifies a unique subset of cases that resemble primary mediastinal large B-cell lymphoma
Source: Blood Cancer J. 2019 Aug 30;9(9):73. doi: 10.1038/s41408-019-0233-5 (PMC6717207; doi:10.1038/s41408-019-0233-5)
Supplement: Supplementary file 1 — Supplementary Material [file 41408_2019_233_MOESM1_ESM.doc]

***Amplification of 9p24.1 in Diffuse Large B-Cell Lymphoma Identifies a Unique Subset of Cases that Resemble Primary Mediastinal Large B-cell Lymphoma***

Wang and Wenzl *et al*, 2019

**Supplemental Tables**

**Supplemental Table 1. Baseline Clinical Characteristics of the 199** DLBCL Patients

|  | **Number** | **%** |
| --- | --- | --- |
| **Age** |  |  |
| ≤60 | 74 | 37.2 |
| >60 | 125 | 62.8 |
| **Sex** |  |  |
| Male | 121 | 60.8 |
| Female | 78 | 39.2 |
| **ECOG PS** |  |  |
| <2 | 174 | 87.4 |
| ≥2 | 24 | 12.1 |
| Missing | 1 | 0.5 |
| **LDH** |  |  |
| Normal | 86 | 43.2 |
| Elevated | 90 | 45.2 |
| Missing | 23 | 11.6 |
| **Extranodal Sites** |  |  |
| ≤1 | 161 | 80.9 |
| >1 | 38 | 19.1 |
| **Ann Arbor Stage** |  |  |
| I-II | 78 | 39.2 |
| III-IV | 121 | 60.8 |
| **IPI Score** |  |  |
| 0-1 | 63 | 31.7 |
| 2 | 55 | 27.6 |
| 3 | 48 | 24.1 |
| 4-5 | 18 | 9.0 |
| Missing | 15 | 7.5 |
| **Cell of Origin** |  |  |
| GCB | 104 | 52.3 |
| ABC/Non-GCB | 60 | 30.2 |
| Unclassifiable | 15 | 7.5 |
| Missing | 20 | 10.1 |

**Supplemental Table 2. Summary of DLBCL Cases with a 9p24.1 CNA**

| **Case #** | **9p24.1 CNA by WES/OncoScan** | **9p24.1 FISH Result** | **Final CNA Call For Clinical Analysis** | **PD-L1 expression by IHC*** | **PD-L1 expression by RNASeq^†^** | **PD-L2 expression by RNASeq^†^** | **JAK2 expression by RNASeq^†^** | **GEP Data Available** |
| --- | --- | --- | --- | --- | --- | --- | --- | --- |
| 106 | Amplification | Amplification | Amplification | 90%; 3/3 | 6.219 | 5.407 | 5.235 | Yes |
| 120 | Amplification | Amplification | Amplification | 90%; 3/3 | NA | NA | NA |  |
| 142 | Amplification | Amplification | Amplification | 90%; 3/3 | 6.987 | 7.616 | 7.229 | Yes |
| 186 | Amplification | Amplification | Amplification | 10%; 3/3 | NA | NA | NA |  |
| 62 | Amplification | NA | Amplification | NA | 6.187 | 5.423 | 6.442 | Yes |
| 192 | Amplification | NA | Amplification | NA | NA | NA | NA |  |
| 126 | Gain | Amplification | Amplification | 90%; 3/3 | NA | NA | NA |  |
| 52 | Gain | Gain | Gain | 10%; 2/3 | NA | NA | NA |  |
| 55 | Gain | Gain | Gain | 50%; 3/3 | NA | NA | NA |  |
| 53 | Gain | Polysomy | Gain | 0%; 0/3 | NA | NA | NA |  |
| 90 | Gain | Polysomy | Gain | NA | NA | NA | NA |  |
| 8 | Gain | NA | Gain | NA | 1.958 | 2.87 | 4.042 | Yes |
| 10 | Gain | NA | Gain | NA | NA | NA | NA | Yes |
| 11 | Gain | NA | Gain | NA | 3.273 | 4.131 | 4.611 | Yes |
| 30 | Gain | NA | Gain | NA | NA | NA | NA |  |
| 82 | Gain | NA | Gain | NA | NA | NA | NA |  |
| 85 | Gain | NA | Gain | 0%; 0/3 | NA | NA | NA |  |
| 118 | Gain | NA | Gain | NA | NA | NA | NA |  |
| 123 | Gain | NA | Gain | NA | NA | NA | NA |  |
| 127 | Gain | NA | Gain | NA | NA | NA | NA |  |

* Percentage positive and intensity; † Log_2_RPKM.

**Supplemental Figures**

**Supplemental Figure 1. Illustration of FISH probes used for detecting 9p24.1 copy number.**

**
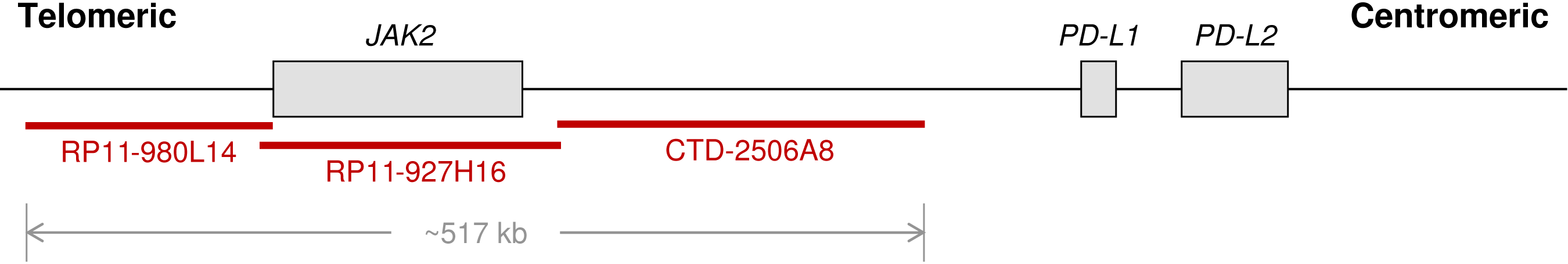
**

Home-brew bacterial artificial chromosome clones RP11-980L14, RP11-927H16 and CTD-2506A8 covering the *JAK2* locus labeled with SpectrumOrange dUTP (Abbott Molecular, Chicago, IL, USA) and chromosome 9 centromere labeled with SpectrumGreen dUTP (Abbott Molecular, Chicago, IL, USA) were used as target and control probes, respectively.

**Supplemental Figure 2. Correlation of WES and OncoScan**

**
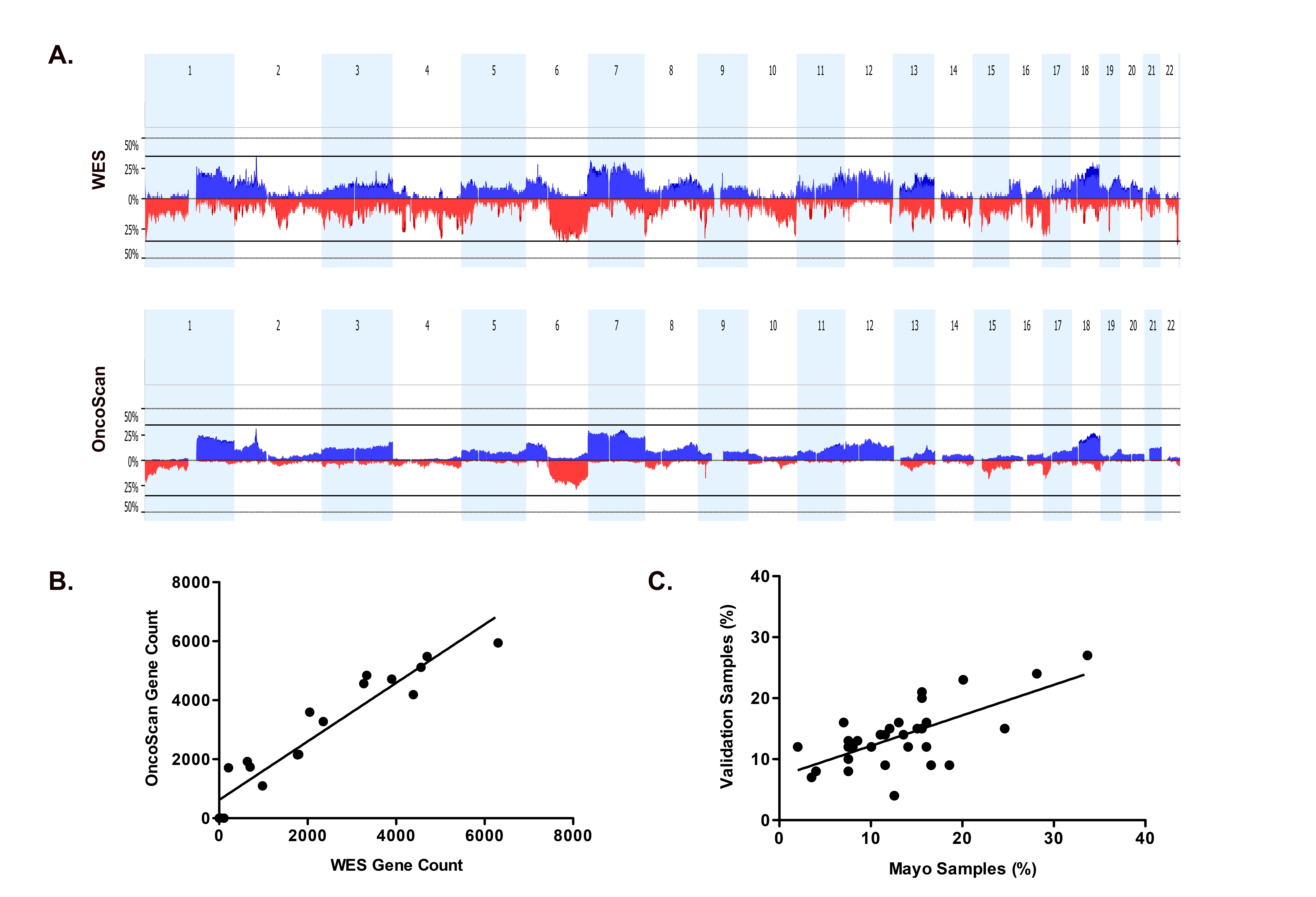
**

A. Frequency of copy number alterations in DLBCL patients analyzed by whole exome sequencing (upper panel, n=57) or OncoScan (lower panel, n=142). B. Comparison of the overall gene level CNAs determine using patient matched WES and OncoScan data (n=19, R^2^=0.90, *P*<0.001). C. Frequency of CNAs (n=31) in our Mayo cohort (n=199) compared with those published by Chapuy *et al* (n=304)^1^ (R^2^=0.457, *P*<0.003).

**Supplemental Figure 3. Comparison of 9p24.1 CNA size.**


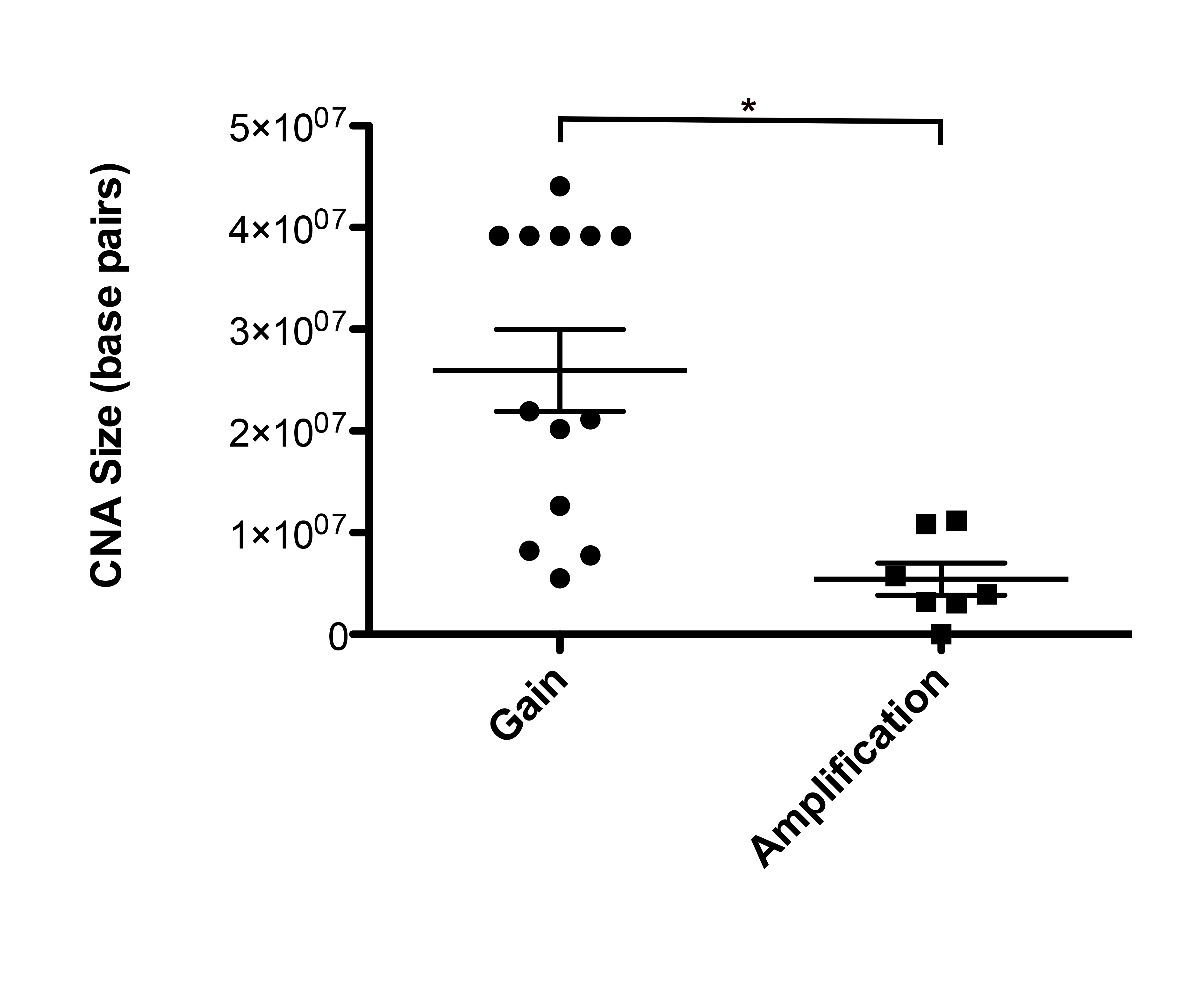


Using Nexus Copy Number software we analyzed the size of the CNA in 9p24.1 gain (n=13) and amplification (n=7) cases. All cases with an amplification had a focal gain (<1.5x10^7^ base pairs) that was significantly smaller in size compared to the gain cases (* *P*<0.01).

**References**

1. Chapuy B, Stewart C, Dunford AJ, et al. Molecular subtypes of diffuse large B cell lymphoma are associated with distinct pathogenic mechanisms and outcomes*. Nat M*ed. 2018;24(5):679-690.
